# Predictable implications of random photon absorption for photoreceptors' gain control

**DOI:** 10.1186/1471-2202-16-S1-P112

**Published:** 2015-12-18

**Authors:** Zhuoyi Song, Yu Zhou, Mikko Juusola

**Affiliations:** 1 Centre for Mathematics, Physics and Engineering in the Life Sciences and Experimental Biology (CoMPLEX), University College London, London, WC1E 6BT, UK; 2 School of Computing, Engineering and Physical Sciences, University of Central Lancashire, PR1 2HE, UK; 3 Department of Biomedical Science, University of Sheffield, S10 2TN, UK; 4 State Key Laboratory of Cognitive Neuroscience and Learning, Beijing Normal University, Beijing 100875, China

## 

Light intensities change enormously in terrestrial environments, from murky starlit night to scorching daylight [1]. Photoreceptors of land animals have evolved with specialized photon absorption structures and adaptive mechanisms to cope with this large input range. They can effectively encode the vastly varying light intensities to macroscopic voltage responses, capturing the temporal structure of natural contrast changes within their limited output range. However, if photoreceptors were purely photon counters, counting every single photon that hits them, their outputs would readily saturate at bright daylight. To lessen this problem, it has been suggested that sublinear summation in quantum bump production (*quantum-gain nonlinearity*) may reduce the bump/photon gain at the instances when multiple photons hit the same photon-sampling-unit (multi-photon-hits) [2]. Here, we quantify the contribution of this *nonlinearity *to light adaptation, using a *Random Photon Absorption Model *for microvillar photoreceptors. We show that the quantum-gain nonlinearity affects only marginally (≤ 1%) photoreceptors with many microvilli, such as those of flies. This is because, with tens of thousands of photon-sampling-units, the probability of multiple photons hitting on any one of them simultaneously is very low. However, this nonlinear mechanism is predicted to affect a photoreceptor's encoding more, if the cell has fewer microvilli, especially when it faces a bright daylight environment.
